# How goats avoid ingesting noxious insects while feeding

**DOI:** 10.1038/s41598-017-14940-6

**Published:** 2017-11-01

**Authors:** Tali S. Berman, Matan Ben-Ari, Tzach A. Glasser, Moshe Gish, Moshe Inbar

**Affiliations:** 1 Department of Evolutionary and Environmental Biology, University of Haifa, Haifa, 3498838 Israel; 2 Ramat Hanadiv Nature Park, POB 325 Zikhron, Ya’akov, 30900 Israel

## Abstract

As mammalian herbivores feed, they often encounter noxious insects on plants. It is unknown how they handle such insects. We experimentally examined the behavioural responses of goats to the noxious spring-webworm (*Ocnogyna loewii*), and manipulated their sensory perception to reveal the process of insect detection. Goats did not avoid plants with webworms, demonstrating a remarkable ability to sort them apart from the plant (98% of webworms survived). Initial detection of webworms involved tactile stimulation, done by repeatedly touching the leaves with the muzzle. This enabled them to pick webworm-free leaves. If the goats picked up leaves with a webworm, they shook or discarded the leaf. They spat out webworms that entered their mouths, after detecting them by touch and taste. By using their keen senses and efficient behaviours, goats are able to feed while accurately excluding insects. These findings highlight the importance of direct interactions between mammalian herbivores and insects.

## Introduction

Due to their size and polyphagous nature, mammalian herbivores (MH) induce changes in the abundance, distribution, phenology, architecture and chemistry of plants in their habitats. These changes can indirectly (plant-mediated) affect insect herbivores (IH) residing on the plants. Yet, MH and IH may also frequently interact directly, for example via ingestion. While indirect interactions between them have been extensively studied^1–3^, direct interactions have received little attention^4^.

MH consume large quantities of plant material^5^. Since IH are ubiquitous on various plants^6^, it is reasonable to assume that they will be directly harmed by feeding MH. Less mobile or sessile IH should face a greater threat of being ingested by MH^7,8^. While evidence of ingestion of IH by MH is scarce, the few existing reports indicate that it might be common and important^9–11^. Another indication for the importance of direct interactions between MH and IH is inferred from the recent discovery that certain aphid species and their coccinellid beetle predators can escape ingestion by recognizing the humid and warm breath typical of MH, immediately dropping off the plant^12,13^. IH can reduce the risk of ingestion by selecting plant parts that are less accessible to MH^9,14^, or by inducing repulsive odors^15^. Nonetheless, it is currently unclear whether MH are attracted, deterred or indifferent to the presence of IH on their food plants^4^.

Ingestion of noxious or unpalatable IH may harm MH. For example, blister beetles (Meloidae) contain cantharidin, which is toxic for MH^16,17^. Eastern tent caterpillars (*Malacosoma americanum*) caused teratogenic diseases in horses that ingested them while grazing^18^. Sheep that grazed on alfalfa infested with aphids and ladybugs suffered from skin lesions and edema^19^. MH diseases may be vectored by IH and transmitted to MH via direct ingestion^20,21^. The ability to avoid ingestion of noxious IH may therefore be critical for MH. On the other hand, harmless IH may provide supplemental proteins to MH.

While many studies examined the ability of MH to discriminate between different plants, there is no data on MH ability to detect noxious IH and avoid them. Ungulates, which are usually polyphagous feeders, are capable of choosing specific plant parts with their flexible lips and tongue^22,23^. They select food using their senses of sight, smell, touch and taste. Perception of brightness, colour and shape allows them to evaluate plants without physical contact^23^. Smell is important for discriminating between different plants^24^. When contact is made, touch allows them to inspect a plant’s texture^25^ and finally tasting allows them to evaluate food properties before ingestion^26^. Generally, sight is considered to be less important than smell, touch and taste in food selection^27^. To the best of our knowledge, behavioural features of food selection in ungulates have not been examined.

Since ingesting of noxious IH might have grave effects on ungulates, we hypothesized that they have the ability to detect and avoid it. Here, we examined how goats respond to the spring webworm (*Ocnogyna loewii*, Lepidoptera, henceforth “webworms”) on their food plants. The polyphagous nature of these two herbivore species and their abundance in Mediterranean-type habitats^28,29^ makes them a convenient model system for studying this hypothesis.

In a preliminary study, we observed numerous encounters between grazing goats and webworms. Surprisingly, the goats did not avoid feeding on plants inhabited by several webworms, however, they never ingested them. Accordingly, we investigated the manner by which goats detect webworms and avoid their ingestion. Specifically, we outlined three main questions: (1) Is goat behaviour while feeding affected by the presence of webworms on their food plants? (2) Does goat feeding affect webworm survival? (3) What sensory modalities do goats use for detecting webworms on their food plants?

## Results

### Goat response to caterpillars on their food plants

**Figure 1:**
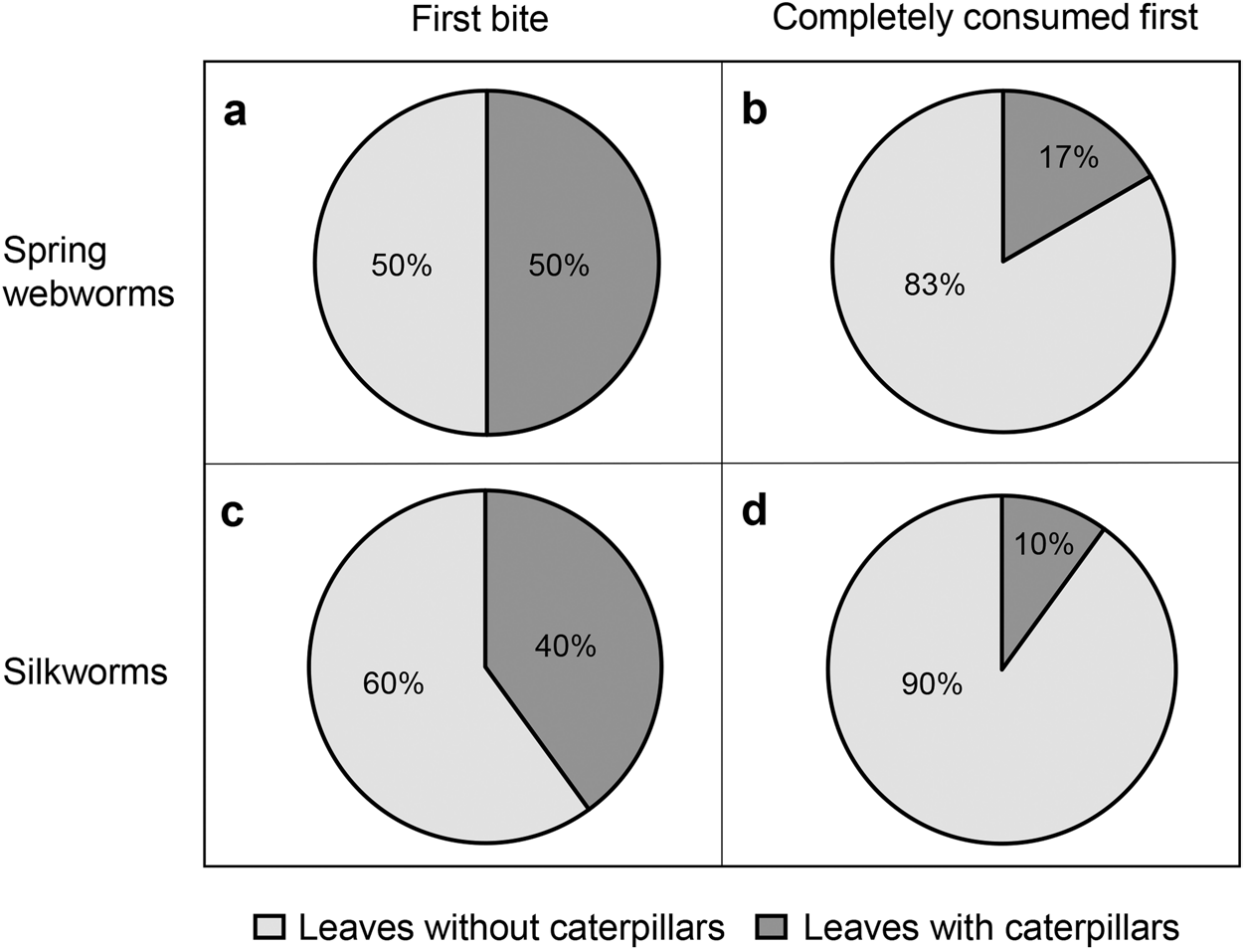
The effect of webworms and silkworms on goat feeding preferences. (**a**,**c**) First bite, the bowl that was eaten from first (webworms: no statistics were performed since the number of goats feeding on each treatment was equal, silkworms: chi square, χ^2^_1_ = 0.4, *P* = 0.527). (**b**,**d**) The bowl that was completely consumed first (webworms: chi square, χ^2^_1_ = 5.33, *P* = 0.021, silkworms: chi square, χ^2^_1_ = 6.4, *P* = 0.011). N = 15 goats in webworm trials and N = 10 goats in silkworm trials.

In order to examine the goats’ initial reaction to IH on their food plants, we presented them with a choice between plain leaves (“control leaves”) and leaves with caterpillars in plastic bowls. Since webworms are spiny caterpillars, we also examined the goats’ initial response to a smooth caterpillar, the silkworm (*Bombyx mori*, leaves with silkworms = “silkworm leaves”; leaves with webworms = “webworm leaves”). We performed this experiment with low and high caterpillar densities. The presence of caterpillars on the leaves did not deter the goats, as they readily fed on them while avoiding caterpillar ingestion. The goats initiated feeding (the bowl from which they started eating first, henceforth “first bite”) from webworm and control leaves equally (Fig. 1a), but finished the control leaves first (Fig. 1b). A similar (not significant) trend was found with the low webworm density (first bite: chi square, χ^2^_1_ = 0.067, *P* = 0.796, the bowl completely consumed first: chi square, χ^2^_1_ = 3.267, *P* = 0.071). Similarly, the goats’ first bite was not affected by the presence of silkworms and they finished consuming the control leaves first (Fig. 1c,d, low silkworm density: first bite: chi square, χ^2^_1_ = 1.6, *P* = 0.206, the bowl completely consumed first: chi square, χ^2^_1_ = 6.4, *P* = 0.011). Once the goats had completely consumed the control leaves, they proceeded to feed on the caterpillar leaves. Remarkably, they did not ingest the caterpillars and over 98% of both species remained intact (Table 1). The remaining caterpillars (2% and less) were injured or lost (were not ingested but could not be found).

**Table 1:** The effect of caterpillar presence on goat feeding.

Caterpillar species	High density of caterpillars					Low density of caterpillars				
	Remaining weight of leaves (%)		Feeding rate (g/s) ± SE		Surviving caterpillars/total	Remaining weight of leaves (%)		Feeding rate (g/s) ± SE		Surviving caterpillars/total
	Without caterpillars	With caterpillars	Without caterpillars	With caterpillars		Without caterpillars	With caterpillars	Without caterpillars	With caterpillars	
Spring Webworms	0	24	1.449 ± 0.145	1.057 ± 0.187	177/180	0	8	1.42 ± 0.214	1.62 ± 0.172	75/75
Silkworms	0	39	0.9 ± 0.07	0.2 ± 0.05	149/150	0	36	1.16 ± 0.09	0.67 ± 0.109	49/50

The goats’ feeding rate (g/s) was not affected by webworms or their density (low density: paired t-test, t_14_ = 0.834, *P* = 0.418, high density: paired t-test, t_11_ = −1.728, *P* = 0.112), yet it dropped in the silkworm trials (low density: paired t-test, t_9_ = −4.155, *P* = 0.002, high density: paired t-test, t_9_ = −7.559, *P* < 0.001). Caterpillar-free leaves were always completely consumed. Webworm trials included 15 and 12 goats for low and high caterpillar densities respectively, and silkworm trials included 10 goats for both caterpillar densities. ± indicate S.E.M.

While the goats consumed all control leaves, they left a considerable amount of caterpillar leaves (Table 1). The goats feeding rate (g/s) was not affected by the presence of webworms, yet dropped in silkworm trials (Table 1).

### Goat behaviour while feeding on plants with webworms

In order to examine how goats avoid ingesting webworms, we analysed their behaviours in non-choice trials while feeding on leaves with and without webworms. Behaviour frequencies were defined as the number of times they appeared per minute. The goats never ingested webworms while feeding (Supplementary movie 1) and performed a series of explicit behaviours to avoid ingestion:

#### Behaviour prior to feeding

**Figure 2:**
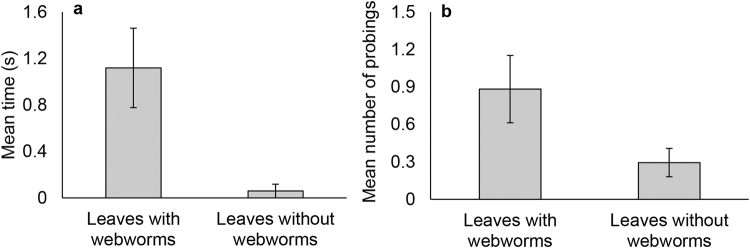
The goats’ behaviour from the moment they approached the leaves (with and without webworms) until they began feeding. (**a**) The time from the goats’ first approach to their first bite (Wilcoxon signed-rank test, Z = −2.871, *P* = 0.004) (**b**) Number of probings (touching the leaves with their muzzles) before feeding (Wilcoxon signed-rank test, Z = −2.308, *P* = 0.021). Bars show means ± S.E.M. N = 17 goats.

The time that passed from the moment the goats approached the webworm leaves until they started feeding was 19 times longer compared to control leaves (Fig. 2a, Wilcoxon signed-rank test, Z = −2.871, *P* = 0.004). This occurred since the goats spent time repeatedly touching the leaves with their muzzles, “probing” (Supplementary movie 2). Each probe lasted about a second and occurred at least once before actual feeding began. The number of probings was nearly three times higher in webworm leaves than the control (Fig. 2b, Wilcoxon signed-rank test, Z = −2.308, *P* = 0.021). The goats did not start feeding and continued probing the leaves as long as their muzzle encountered a webworm. Hence, the number of probings on webworm leaves was directly proportional to the number of webworms contacted by the goats’ muzzle (Spearman’s rho, since the data did not follow a normal distribution, R = 0.828, *P* < 0.001).

#### Behaviour while feeding

**Figure 3:**
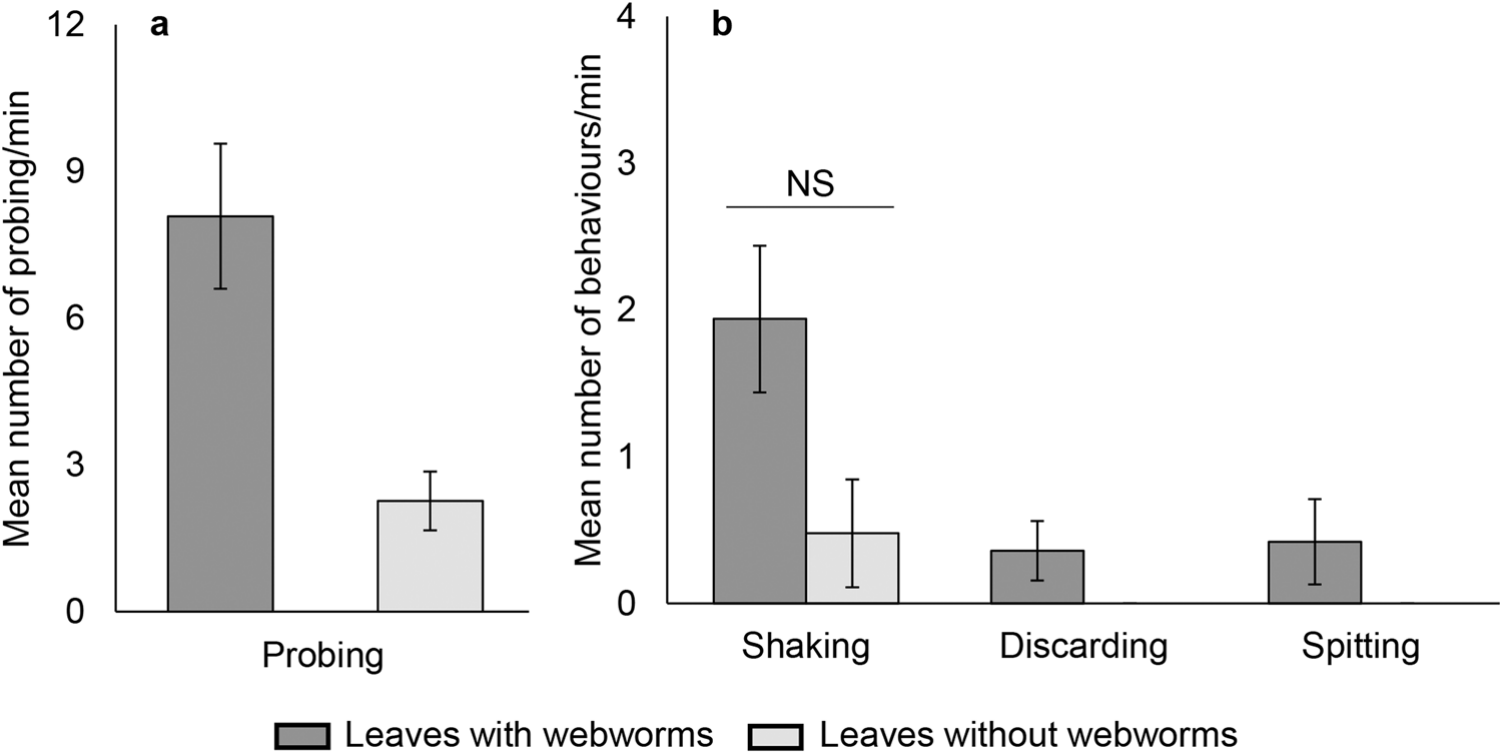
Frequencies of goat behaviour while feeding on leaves with and without webworms. (**a**) Probing (Wilcoxon signed-rank test, Z = −3.206, *P* = 0.001) (**b**) Additional behaviours (Shaking: Wilcoxon signed-rank test, Z = −1.889, *P* = 0.058). Spitting and discarding appeared only when webworms were present (no statistical analyses required). The behaviours were statistically analysed separately. Bars show means ± S.E.M.

We defined three additional behaviours that appeared more frequently or exclusively when the goats fed on webworm leaves (Fig. 3; Supplementary movie 2): 1) “Shaking”- before chewing the leaf, the goat moved its head up and down on a vertical axis, between one to five times. This fast motion lasted up to three seconds and was followed by leaf ingestion. 2) “Discarding”- the goat dropped or tossed the leaves without ingestion. Discarding involved direct contact between the webworm and the goat’s muzzle. 3) “Spitting”- occurred mostly when webworms entered the goat’s mouth and was used to eject the webworms. Probing was the most prominent behaviour when webworms were present (Fig. 3a) followed by shaking (Fig. 3b), yet the number of shakings per minute between webworm and control leaves was insignificant (*P* = 0.058). Discarding and spitting appeared only when consuming webworm leaves. The goats efficiently used these cascades of behaviours, before and while feeding, to avoid webworm ingestion. Overall, probing enabled them to mostly pick webworm-free leaves, leaving behind 85% of the webworms (289 of 340). The goats then attempted to dislodge webworms picked up with the leaves by shaking them off (44 of 51) or discarding them (3 of 51). Remarkably only 1% of all webworms (4 of 340) entered the goats’ mouths and were spat out. Nearly all webworms survived (3 of 340 were injured or lost).

#### Goat behaviour while feeding on leaves with tied webworms

**Figure 4:**
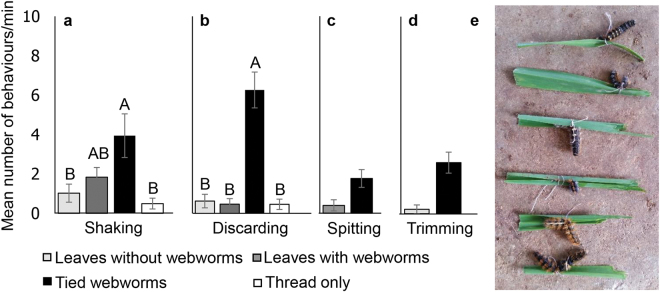
The effect of tied webworms on goat behaviour. The frequency of behaviours employed by the goats while feeding on leaves without webworms, leaves with webworms, tied webworms (tied to the leaves with sewing thread), and leaves with thread only. (**a**) Shaking (Friedman test, χ^2^_3_ = 18.674, *P* < 0.001) (**b**) Discarding (Friedman test, χ^2^_3_ = 35.976, *P* < 0.001) (**c**) Spitting (Wilcoxon signed-rank test, Z = −2.389, *P* = 0.017) (**d**) Trimming (Wilcoxon signed-rank test, Z = −3.163, p = 0.002). Different letters above bars indicate significant differences. Bars indicate means ± S.E.M. (**e**) The goats cut the leaves with tied webworms (trimming side on the right). The webworms always remained undamaged during this behaviour. N = 18 goats.

In order to examine the goats’ behaviour while feeding on leaves inhabited by IH that cannot be easily removed (i.e. pupae), we tied dead webworms to the leaves using sewing thread (for easier handling). We then compared the goats’ behaviour while feeding on control leaves, webworm leaves (live), leaves with tied webworms and leaves with sewing thread alone, in non-choice trials. When the goats encountered tied webworms, all previously observed behaviours appeared (Fig. 4a–c), but their frequency increased as discarding became the most prominent behaviour. In response to webworm immobility, the goats exhibited a new behaviour that was not recorded before, “trimming” (Fig. 4d,e). The goat ate the leaves until its lips contacted the tied webworm, then it cut the remaining leaves with the (undamaged) webworm and dropped it (Supplementary movie 2). Consequently, 83% of tied webworms (224 of 270) remained intact, compared to 99% of free webworms (267 of 270, Wilcoxon signed-rank test, Z = −3.438, *P* = 0.001). The goats consumed 54% of leaves with tied webworms, 82% of webworm leaves, 94% of control leaves and 97% of leaves with thread only (Friedman test, χ^2^_3_ = 24.490, *P* < 0.001).

In an additional control, we tested the goats’ reaction to dead and live webworms and found they reacted similarly (Shaking: Wilcoxon signed-rank test, Z = −0.525, *P* = 0.6, Discarding: Wilcoxon signed-rank test, Z = −1.352, *P* = 0.176), however, spitting appeared only in the live webworm treatment (0.39 spits per minute). This occurred since frozen webworms do not cling to leaves and fall off as soon as they are picked up with the leaves, minimizing the possibility of webworms entering the goats’ mouths. Nevertheless, frozen webworms did not alter goat feeding behaviour.

### Sensory modalities involved in the goat’s ability to detect webworms

#### The role of sight in detecting webworms

We tested the goats’ ability to detect webworms using sight by covering their eyes with a blindfold. We then presented the goats with a choice between food pellets with and without webworms. Blindfolded goats initiated feeding from both treatments equally (chi square, χ^2^_1_ = 0.091, *P* = 0.763), however, all goats finished consuming the pellets without webworms first. The blindfold did not interfere with the goats’ ability to avoid webworm ingestion (one webworm out of 165 was not found) and they consumed 75% and 100% of pellets with and without webworms respectively.

#### The role of smell in detecting webworms

We tested the goats’ ability to detect webworms using smell by presenting them with a choice between food pellets with and without webworms when covered by perforated lids. The goats were able to smell the content of the bowls without seeing or touching it. The goats did not show a preference for either treatment when covered, as indicated by the similar sniffing time (paired t-test, t_15_ = −1.115, *P* = 0.282). In an additional experiment, we masked the goats’ sense of smell by applying a mentholated topical ointment to their nostrils. The ointment did not interfere with the goats’ ability to avoid webworm ingestion (all webworms survived) and they consumed 95% and 75% of control and webworm leaves respectively, both when their sense of smell was normal and when it was masked (Friedman test, χ^2^_3_ = 10.984, *P* = 0.012).

#### The role of touch in detecting webworms

**Figure 5:**
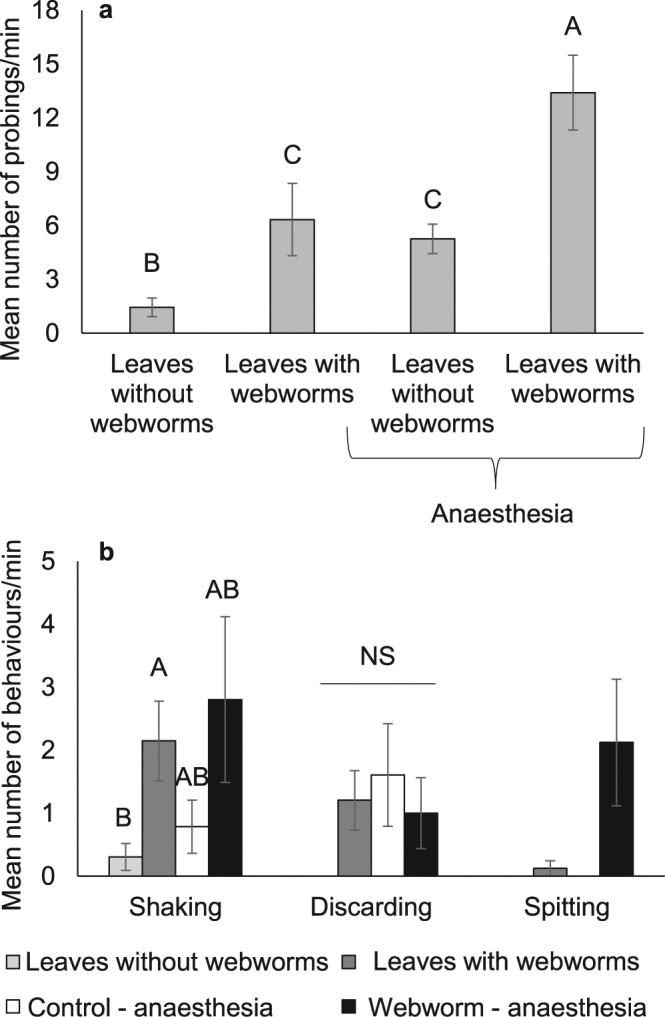
The effect of impaired tactile sensation on the goats’ feeding behaviour. Local anaesthesia was applied to the outer and inner lip area (**a**) Probing (Friedman test, χ^2^_3_ = 22.956, *P* < 0.001). (**b**,**c**,**d**) Additional behaviours (Shaking: Friedman test, χ^2^_3_ = 8.133, *P* = 0.043, Discarding: Friedman test, χ^2^_3_ = 0.6, *P* = 0.741, Spitting: Wilcoxon signed-rank test, Z = −1.992, *P* = 0.046). Different letters above bars indicate significant differences. Bars indicate means ± S.E.M. N = 14 goats.

We tested the goats’ ability to detect webworms using touch by applying a local anaesthetic to their outer and inner lips, impairing their ability to perceive tactile stimulation. Probing increased by nine-fold when the sense of touch was impaired (Fig. 5a) and it was most eminent when webworms were present. The local anaesthesia did not affect the number of shakings and discardings per minute in webworm leaves, however, spitting events were 17 times higher in touch-impaired goats (Fig. 5b). Despite entering the goats’ mouths (causing increased spiting events), 208 of 210 webworms survived (two were injured or lost).

#### The role of taste in detecting webworms

We tested the goats’ ability to detect webworms using taste by presenting them with a choice between plain food pellets and pellets with webworm powder (lyophilized and ground webworms). This also prevented the goats from detecting the webworms by sight or touch. The goats used their sense of taste to detect and avoid pellets mixed with webworm powder. Most of the goats (14 of 15) tasted the pellets before choosing to avoid them. They consumed 92% of the control pellets, but only 10% of the pellets with webworm powder (Wilcoxon signed-rank test, Z = −3.411, *P* < 0.001).

## Discussion

We show here that goats are capable of detecting IH and possess several behaviours that allow them to avoid IH ingestion while still feeding on part of the plant. The fact that goats do not avoid leaves with caterpillars (but never ingested them) implies that they rely on their highly efficient senses and fine behaviours to continue feeding safely.

As selective feeders, goats routinely discriminate between edible and unpalatable or toxic plants^30^. We found that goats do not avoid plants with noxious webworms due to their ability to sort them apart from the leaves. Nonetheless, they may eventually give up on plants with caterpillars (Table 1), if sorting out the leaves takes too long. Although the goats’ initial choice was not affected by webworms, they eventually preferred consuming control leaves that were easier to handle. The optimal foraging theory predicts that animals will select food items that yield the most energy compared to handling time and should consume less profitable food items when the abundance of profitable food items declines^31^. Hence, while grazing in a competitive environment, it is important for MH to be able to utilizable at least part of the plants despite the presence of IH.

Studies on the selective feeding behaviour of goats have so far focused mainly on dietary preferences^32^ and not on the specific behaviours they use to select their food. Here, we reveal the behaviours goats possess to avoid consuming IH. Probing (the most frequent behaviour) enabled the goats to pick webworm-free parts, leaving behind 85% of the webworms. Once the goats became aware of the presence of webworms via probing, shaking increased regardless of whether they picked up webworms with the leaves. Since the goats may not know for sure if webworms were picked up, it is worthwhile for them to increase shaking that efficiently dislodges webworms. When shaking was unsuccessful (as with tied webworms), they discarded or trimmed the leaves. If a webworm entered the goats’ mouths, it was spat out mostly undamaged. Ejection of unpalatable or toxic plant parts has been reported in horses^33^ and in goats^34^.

Sessile IH (i.e. pupae) cannot be dislodged. When facing tied webworms, the goats increased their behaviour frequencies. Since shaking was ineffective, spiting events increased (by four-fold). Consequently, discarding became the most prominent behaviour and trimming appeared. The goats seemed to make a great effort in avoiding webworm ingestion, still, they consumed less leaves and several tied webworms were injured (17%) indicting that sessile IH are more vulnerable to MH feeding.

Goat behaviour alone cannot entirely account for the high webworm survival. Some IH species may drop off the plant when escaping danger, such as feeding MH^12,13,35,36^. Certain caterpillar species are able to rapidly escape predators by curling up and rolling off the plant^37^. Webworms may drop off plants and curl up when their plant is picked up by humans and goats (TS Berman personal observations). By shaking the leaves, goats may induce a dropping escape response in IH. Since not all IH are capable of escaping the plant^12,13^, both MH and IH behaviours contribute in reducing IH ingestion.

Ungulate grazing in natural habitats is a complex behaviour. Sight and smell are used by ungulates to evaluate food mostly prior to physical contact, while touch and taste are important once contact with the food is made^26^. We found that goats rely mostly on the sense of touch (and rarely taste) to detect webworms. Touch provides the goats with a fast and accurate way of detection that is essential to avoid the undesirable ingestion of IH. Blindfolded goats managed to avoid webworms, stressing that sight is not important in webworm detection. In addition, if sight were important, the goats would have started eating from the control leaves first (first bite) when given a choice, but they selected leaves without caterpillars only after feeding began. Indeed it seems that sight is less important for ungulates in food selection^38^. Goats, like other ungulates, have a wide field of vision enabling them to detect predators in the distance, yet their visual perception of the space in front of them is rather poor^24^. Grazing ungulates therefore may not be able to see IH, because they are small, camouflaged or obscured by plant parts. Ungulates may also graze during the night when IH are less visible. Hence it is quite clear why goats do not rely on sight to detect IH.

Smell is important for discriminating between plants mostly before ingestion^15^. However, in the absence of negative digestive feedback (poison shyness) it may not always deter ungulates^39,40^. We found that smell is not used by goats to detect webworms, since they did not discriminate between covered control pellets and pellets with webworms, and they detected webworms despite their impaired sense of smell. In addition, they relied on taste (and not smell) to avoid food pellets mixed with webworm powder. It is possible that smell is not reliable since the odour of IH may be similar to that of their host plants^41,42^, or masked by them^43^ making it difficult for ungulates to detect IH based on smell alone.

Grazing ungulates possess a sensitive nervous structure within the epidermis of their muzzles which allows them to select food according to texture^25^. Goats in particular have flexible, sensitive and mobile lips used for picking food items^44,45^. The prevalent probing behaviour (Figs 2 and 3) and their ability to discard and trim leaves as soon as webworms contact their lips, stress that goats depend on touch to detect IH. When we disrupted their ability to sense tactile stimulation with local anaesthesia, probing events increased (Fig. 5). Unlike sight and smell, touch is effective day and night, not influenced by confusing odours, visibility or location on the plant. By inspecting food with their muzzles, ungulates can accurately avoid IH ingestion while still consuming the plant matter.

The acceptance of food once it enters the oral cavity is determined by tactile stimulation and taste. Goats have a sensitive palate used to monitor mechanical properties of food^46^ and they are able to recognize the five basic flavors^26,47^. The fact that goats immediately spat out webworms that entered their mouths (mostly undamaged) and rejected pellets mixed with webworm powder only after tasting them, clearly demonstrates the importance of taste in preventing webworm ingestion. The goats’ ability to detect webworms within their mouths before chewing begins may reduce their exposure to toxic hemolymph^48,49^. Ungulates cannot risk ingesting noxious IH^16,18^, therefore, in the rare case that IH are not successfully dislodged and enter their mouths while feeding, taste provides an effective last resort to prevent IH ingestion.

Combining information received from several sensory modalities can help animals make better decisions and reduce uncertainty^50,51^. However, goats rely mostly on a single sense (touch and rarely taste) to detect webworms. By relying on the quick and simple solution of touch, goats succeed in feeding on plants with IH that may not always stand out visually and chemically in nature. The senses used to detect IH may likely change according to the IH and plant species involved, their location and abiotic conditions.

**Figure 6:**
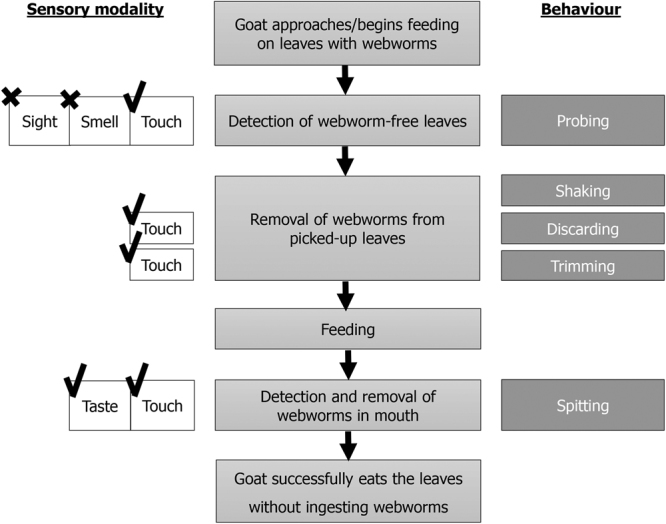
A scheme of the chain of events that occurs when a goat feeds on leaves with webworms. The sensory modalities used by the goats to detect the webworms on the leaves are depicted on the left and the behaviours employed to dislodge them are on the right. Note that goats do not avoid eating leaves with webworms.

When a goat approaches a plant inhabited by webworms, it uses its senses and a cascade of efficient behaviours to detect and remove them while feeding, as summarized in Fig. 6 (see similar behaviours with silkworms, Supplementary Movie 3). Initial detection involves tactile stimulation, obtained by the goat probing the plant with its muzzle and enabling it to pick webworm-free parts. Webworms picked up are shaken off the plant. When shaking is unsuccessful, the goat relies on direct contact to detect and dislodge webworms, by discarding or trimming the leaves. Finally, if a webworm enters its mouth, the goat detects it using touch and taste and spits it out mostly undamaged. The reliability of the goat’s senses (touch and taste) and behaviours enable it to continue feeding safely from plants despite the presence of IH. It is likely that these behaviours are also routinely used by goats while selecting or avoiding specific plant parts regardless of IH presence.

The caterpillars tested in this study were conspicuous and unpalatable to goats that consistently avoided their ingestion. The reactions of goats to other IH species is likely to be influenced by their palatability and toxicity. For example, MH may ingest small relatively benign insects, such as aphids and leaf miners^8,12^. It is very likely that the assemblages of IH species present on plants will affect the behaviours and senses employed by the goats and final occurrence of IH ingestion.

Ungulates have a great influence on the composition and functioning of vegetation. Past studies did not consider the possible effects of IH on ungulate feeding dynamics. We found that goat food consumption and feeding behaviour depended on the caterpillar species, density and mobility (Table 1 and Fig. 4). This finding emphasizes the importance of integrating the outcome of the direct trophic interactions between ungulates and IH in future herbivory studies.

Direct trophic interactions between MH and IH have rarely been studied^4^. Little is known about the ecological consequences of these interactions and the behavioural and physiological processes behind them, even though MH and IH are of great importance in terrestrial ecosystems^6,52^. Our research reveals that ungulates are aware of IH on their food plants and that they can handle them. Ungulate feeding behaviour is complex and their preferences depend on the characteristics of the food, which may be influenced by the presence of IH. The fact that ungulates have developed behavioural mechanisms to avoid IH ingestion points to the prevalence of these direct interactions and the importance of taking them into account in future studies.

## Materials and Methods

### Study organisms

The goats in this study were part of a herd (mostly females) at Ramat Hanadiv Nature Park, Israel. They forage daily in the natural Mediterranean maquis and receive supplementary food.

Webworms are polyphagous, diurnal caterpillars that are common in Mediterranean-type habitats (especially grasslands). They hatch in spring and the first three instars feed together inside a common web nest. Fourth and fifth instar caterpillars disperse and feed solitarily. In the summer, the webworms pupate in the soil and adults emerge in autumn. Winged adult males locate and mate with wingless females, that later lay their eggs in masses under stones or on the soil surface to complete their univoltine life cycle^29^. During the spring, dense caterpillars populations (1–12 per square meter) can be found especially in grasslands (TS Berman unpublished data). Since spring is also the peak of MH feeding season in the Mediterranean region, it is likely that webworms and grazing MH will frequently encounter one another.

We collected solitary webworms (fourth-fifth instars) from natural populations. Webworms are covered with setae, which can potentially provide tactile stimulation for goats. Therefore, we also examined the goats’ initial response to a smooth (no setae) caterpillar, the silkworm (*Bombyx mori*, fourth-fifth instar), which is easy to maintain and work with. The experiments were approved by the Animal Experimentation Ethics Committee of Israel, approval no. IL 678/16, and all methods were carried out in accordance with relevant guidelines and regulations.

### General experimental design

Barley stems and leaves served as food in webworm trials and mulberry leaves were used in silkworm trials. On the morning of an experiment, we left 20 goats in the pen, feeding on hay. Since not all goats were always cooperative, the number of replicates in each experiment was not consistent (specific numbers appear in each experiment). All experiments were conducted in a 3 × 4 m enclosure within the pen. Each goat was led into the enclosure and was allowed to feed voluntarily.

In the choice experiments, we presented each goat with two adjacent (randomized) plastic bowls (20 cm diameter), placed on a wooden manger. One bowl contained 20 g barley or mulberry leaves (“control leaves”) and the second bowl contained 20 g barley or mulberry leaves with caterpillars (“webworm leaves” or “silkworm leaves” respectively). In the non-choice experiments we presented each goat with one bowl at a time (control or webworm) in randomized order.

Once the experiment ended (when the goat voluntarily finished feeding), we weighed the remaining leaves to calculate “food intake” (% of leaves consumed) and monitored caterpillar fate (intact, injured, ingested or lost- when a caterpillar was not ingested, but could not be found). We determined feeding rate as the weight of leaves consumed divided by feeding duration (g/s). The trials were filmed with a high definition camera (GoPro^©^ Hero 4 black edition, USA). We defined specific behaviours from the videos and determined their frequencies as the number of times they appeared per minute. We tested all data for normality using the Shapiro-Wilk test. Data that did not meet the assumptions of parametric tests were tested with equivalent nonparametric tests (see below). All tests used were two-tailed. Statistical analyses were performed using IBM SPSS software v.20 (specific statistical analyses for each experiment appear hereafter).

### Goat response to caterpillars (webworms and silkworms) on their food plants

In order to study the goats’ initial reaction to IH on their food plants, we presented them with a choice between control and caterpillar leaves with low and high caterpillar densities (5 or 15). We determined which bowl the goat began to eat from first (“first bite”) and which bowl was completely consumed first. We compared these criteria using the Pearson chi-square test. Goat feeding rate (g/s) was analysed using a paired t-test. Since control leaves were always completely consumed first, no statistics were needed. Webworm trials included 15 and 12 goats for low and high caterpillar densities respectively, and silkworm trials included 10 goats for both caterpillar densities.

### Goat behaviour while feeding on plants with webworms

We recorded goat behaviour (N = 17) while feeding on control and webworm leaves (20 to amplify goat behaviour) in non-choice trials. We counted the number of webworms that the goat dislodged by the different behaviours (only 11 of 17 goats picked up leaves with webworms). We compared the frequency of behaviours using the Wilcoxon signed-ranks test (since the data did not follow a normal distribution).

#### Goat behaviour while feeding on leaves with tied webworms

In order to mimic a situation where IH cannot be removed from the plant (i.e. pupae), we tied the webworms to leaves with sewing thread. We used dead webworms (frozen and thawed, 15 per trial) for easier handling. In non-choice trials we compared goat behaviour (N = 18) while feeding on control leaves, webworm leaves, leaves with tied webworms and leaves with sewing thread alone. As an additional control, we tested the goats’ reaction to dead webworms. The frequency of behaviours and food intake data did not did not follow a normal distribution, therefore they were analysed using the Friedman test when more than two treatments were compared (post hoc Wilcoxon signed-rank test, Bonferroni corrected for multiple comparisons) and the Wilcoxon signed-rank test when two treatments were compared (individual goat as the repeated-measures factor).

### Sensory modalities involved in the goat’s ability to detect webworms

#### The role of sight

We covered the goats’ eyes with a soft cloth blindfold (tied at the back of the neck) and led them to the manger. Then we presented the goats (N = 11) with a choice between 30 g food pellets (processed feed) without and with 15 webworms. We compared the “first bite” incidents using the Pearson chi-square test. Since the goats completely consumed all the control pellets first, no statistical analysis was needed.

#### The role of smell

We presented the goats (N = 16) with a choice between 20 g pellets without and with 30 webworms. We then covered the pellets with perforated lids (15 holes of 3 mm diameter per lid), allowing the goats to smell the bowls without seeing or touching the contents for two minutes. The time spent sniffing each bowl was analysed with a paired t-test. In another experiment, the goats’ (N = 15) sense of smell was masked by applying a mentholated topical ointment (VapoCal^©^, STH health care, Haifa, Israel) to their nostrils. Such ointments, as Vicks^®^ VapoRub™, have been used on ruminants for similar purposes^53^. Webworm survival (15 per trial) and food intake were measured in non-choice trials (webworm and control) with and without the ointment. Food intake was analysed using the Friedman test since the data did not follow a normal distribution (individual goat as the repeated-measures factor).

#### The role of touch

We examined goat behaviour in non-choice trials (control and webworm, 15 per trial) with and without lip anaesthesia. We impaired the goats’ (N = 14) ability to perceive tactile stimulation by applying a local anaesthetic (HurriCaine^©^ topical anaesthetic gel, 20% benzocaine oral anaesthetic, wild cherry, Beutlich LP pharmaceuticals, Waukegan, USA) to their outer and inner lips. Benzocaine is used for anesthetizing ruminants’ mouths^54^. The frequency of behaviours and food intake data did not follow a normal distribution, therefore they were analysed using the Friedman test when more than two treatments were compared (post hoc Wilcoxon signed-rank test, Bonferroni corrected for multiple comparisons) and the Wilcoxon signed-rank test when only two treatments were compared (with the individual goat as the repeated-measures factor). In addition, we counted the surviving webworms.

#### The role of taste

We gave the goats (N = 15) a choice between 30 g plain pellets and 30 g pellets mixed with webworm powder (lyophilized for 72 hours and ground, 15 per trial). This setup eliminated the goats’ ability to detect webworms by sight or touch. We used the videos to determine whether the goats made their choice based on smell (sniffing without ingesting) or taste (ejecting pellets). We compared the food intake using the Wilcoxon signed-ranks test (since the data did not follow a normal distribution).

### Data availability

The data that support the findings of this study are available from the corresponding author upon reasonable request.

## Electronic supplementary material

Supplementary movie 1

Supplementary movie 2

Supplementary movie 3

Supporting information
