# Supplementary material for: How goats avoid ingesting noxious insects while feeding
Source: Sci Rep. 2017 Nov 1;7:14835. doi: 10.1038/s41598-017-14940-6 (PMC5665868; doi:10.1038/s41598-017-14940-6)
Supplement: Supplementary file 4 — Supporting information [file 41598_2017_14940_MOESM4_ESM.pdf]

Table manners matter: how goats avoid ingesting noxious insects while feeding

Tali S. Berman[a], talisberman@gmail.com

Matan Ben-Ari[a], matbenari@gmail.com

Tzach A. Glasser[b], Tzach@ramathanadiv.org.il

Moshe Gish[a], mozygish@hotmail.com

*Moshe Inbar[a], minbar@research.haifa.ac.il

[a]Department of Evolutionary and Environmental Biology, University of Haifa, Haifa, 3498838, Israel

[b]Ramat Hanadiv Nature Park. POB 325 Zikhron Ya'akov 30900, Israel

*Corresponding Author: Moshe Inbar, Department of Evolutionary and Environmental Biology, University of Haifa, Haifa 3498838, Israel, phone number: + 972 4 8288767, e-mail: minbar@research.haifa.ac.il

Video captions and legends

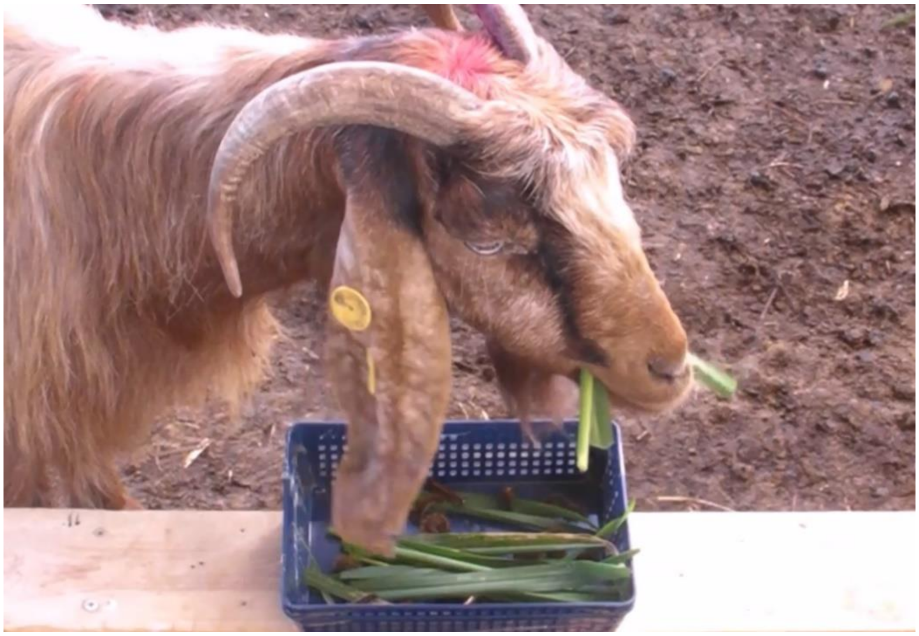

Supplementary movie 1. A feeding goat avoiding the ingestion of numerous webworms. All webworms remain intact. Video speeded by ×3.

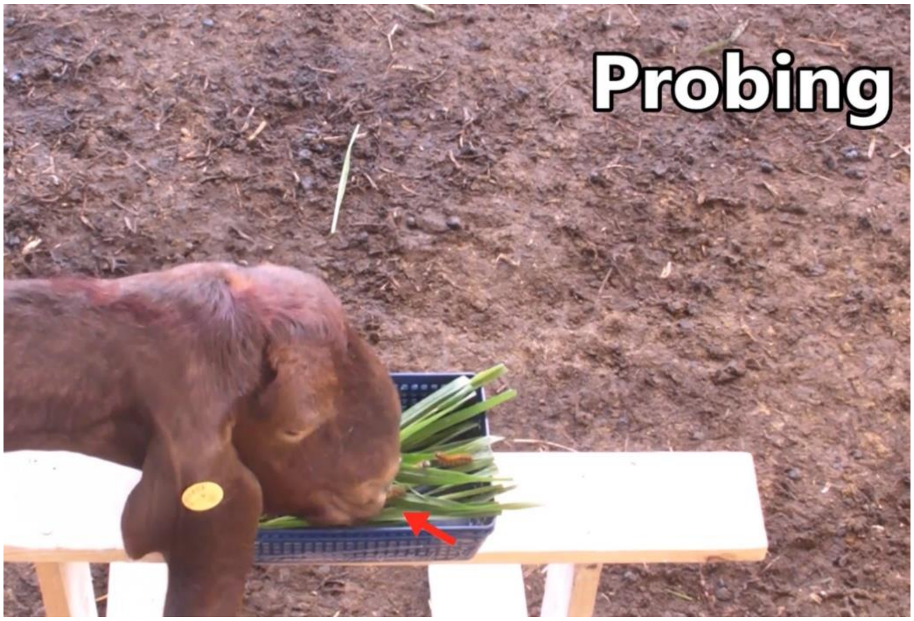

Supplementary movie 2. Goat behaviour while feeding on leaves with webworms (dislodged webworms are marked with red arrows or circles). By repeatedly touching the leaves with their

muzzles (probing), the goats were able to pick webworm-free leave parts. If the goats picked up leaves with a webworm, they shook it off by vertically moving their head up and down. When shaking was unsuccessful, they tossed the leaves (discarding) or ate around the webworm (trimming). Finally, if a webworm entered the goats' mouth, they spat it out mostly undamaged. These efficient behaviours enabled the goats consumed leaves only despite webworm presence. Video speed decreased by ×0.5.

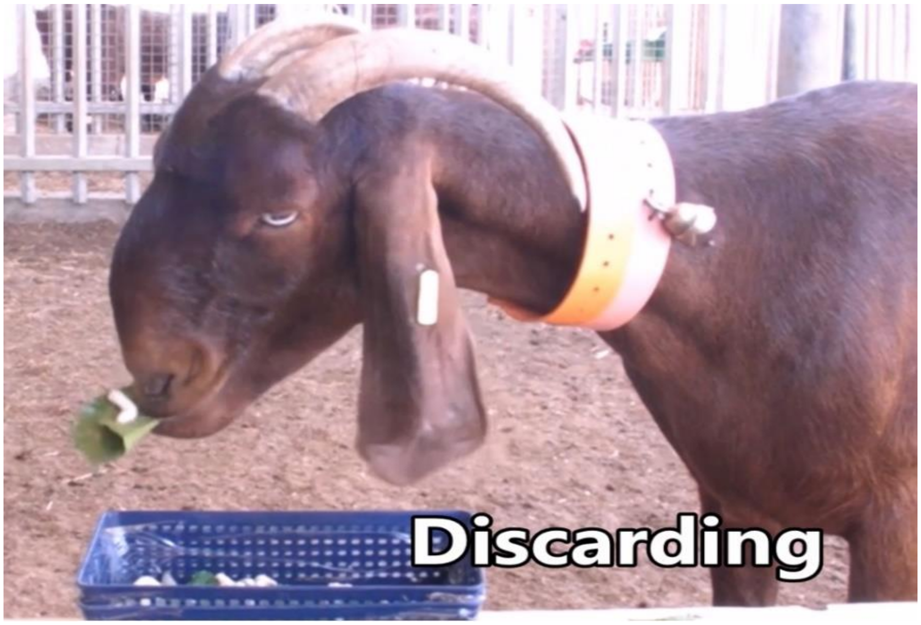

Supplementary movie 3. Goat behaviour while feeding on leaves with silkworms. The behaviours exhibited by the goats are similar to those observed with webworms.
